# Glycolytic alterations as biomarkers in polycystic kidney disease: A study using a *PKD1* knockout model in NRK‐52E rat kidney epithelial cells

**DOI:** 10.14814/phy2.70816

**Published:** 2026-03-19

**Authors:** Ida Kjær Mieritz, Christoffer Laustsen, Esben Axelgaard, Sofie Rahbek Dorset, Rasmus O. Bak, Lotte Bonde Bertelsen

**Affiliations:** ^1^ The MR Research Center, Dept. of Clinical Medicine Aarhus University Aarhus N Denmark; ^2^ Department of Biomedicine Aarhus University Aarhus C Denmark

**Keywords:** CRISPR, hyperpolarization, kidney, MRS, PKD1

## Abstract

Polycystic kidney disease (PKD) is a genetic disorder characterized by the formation of fluid‐filled cysts in the kidneys, often resulting in progressive renal impairment. Mutations in the PKD1 gene represent the predominant genetic cause of autosomal dominant PKD. Here, we investigated how PKD1 knockout affects glycolytic metabolism in NRK‐52E kidney epithelial cells using dynamic nuclear polarization (DNP)‐enhanced magnetic resonance spectroscopy (MRS) with hyperpolarized [1‐^13^C]pyruvate. PKD1 knockout NRK‐52E kidney epithelial cells showed a significantly elevated pyruvate‐to‐lactate conversion as measured by hyperpolarized [1‐^13^C]pyruvate (HP‐MRS) and significantly increased lactate levels in culture medium, accompanied by upregulated lactate dehydrogenase (LDH) gene expression and enzymatic activity. Monocarboxylate transporter (MCT) expression was selectively altered (significant downregulation of MCT2 and MCT3; MCT1 not significantly changed). Pyruvate dehydrogenase (PDH) activity and transcript levels did not differ between groups. These results demonstrate glycolytic reprogramming associated with PKD1 deficiency and support hyperpolarized pyruvate MRS as a sensitive metabolic biomarker for detecting such alterations in real‐time. These findings identify glycolytic remodeling as a robust metabolic consequence of PKD1 loss and demonstrate that HP‐[1‐^13^C]pyruvate MRS provides a flux‐level biomarker suitable for real‐time metabolic characterization.

## Graphical abstract

CRISPR–Cas9–mediated PKD1 knockout in NRK‐52E rat kidney epithelial cells induces glycolytic reprogramming. Hyperpolarized [1‐^13^C]pyruvate magnetic resonance spectroscopy reveals increased pyruvate‐to‐lactate conversion accompanied by elevated lactate dehydrogenase activity and altered monocarboxylate transporter expression. Created in BioRender.

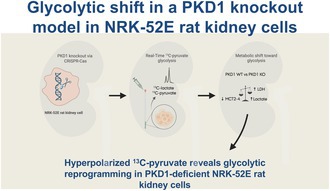

## 1 INTRODUCTION

Polycystic kidney disease (PKD) is a genetic condition characterized by the growth of cysts within the kidneys, which gradually enlarge and impair renal function (Halvorson et al., 2010). This progressive growth compromises renal filtering capabilities, eventually leading to end‐stage renal disease (ESRD), a critical condition requiring dialysis or kidney transplantation for survival (Chapman et al., 2015; Jensen et al., 2016). PKD presents in two primary forms: autosomal dominant PKD (ADPKD) and autosomal recessive PKD (ARPKD). ADPKD, which accounts for the majority of cases, impacts 1 in 500 to 1000 individuals worldwide (Paul & Vanden Heuvel, 2014). It is predominantly caused by mutations in the *PKD1* gene (85% of cases), with the remaining cases linked to *PKD2* mutations (Torres et al., 2007). ARPKD is less common, typically presenting in infancy and associated with additional complications such as biliary tract defects and oligohydramnios (Hildebrandt & Zhou, 2007).

The *PKD1* gene encodes polycystin‐1 (PC1), which forms a functional complex with polycystin‐2 (PC2) encoded by the *PKD2* gene, to regulate cellular mechanosensation and signaling (Qian et al., 1997; Wilson, 2004). Despite extensive investigation, the molecular mechanisms linking PC1/PC2 deficiency to cystogenesis remain incompletely understood. Current evidence implicates disruptions in cell proliferation, fluid transport, apoptosis, and protein localization (Lee, 2016; Reiterova & Tesar, 2022; Zhang et al., 2021). However, conventional imaging and biochemical techniques lack the sensitivity to monitor these dynamic metabolic changes in real time, limiting insight into disease progression and therapeutic response (Ghanem et al., 2024; Harris & Torres, 2009).

While ADPKD cysts most commonly originate from the distal nephron or collecting duct (Torres et al., 2007; Wilson, 2004), we used the NRK‐52E proximal tubular epithelial cell line as a reductionist, mechanistic model. NRK‐52E kidney epithelial cells maintain stable epithelial identity, are highly amenable to CRISPR editing, and avoid differentiation‐related variability. Although this 2D monolayer system does not recapitulate cyst morphology, lumen expansion, or nephron‐segment specificity, it enables controlled interrogation of the direct metabolic effects of PKD1 loss independent of architecture‐ or fluid‐shear–dependent processes. This model therefore complements, rather than replaces, future work in 3D cyst systems and in vivo models.

Hyperpolarized [1‐^13^C]pyruvate Magnetic Resonance Spectroscopy (HP‐MRS) enables dynamic measurement of pyruvate‐to‐lactate conversion and complements static metabolic assays used in PKD research. Unlike static metabolite assays, HP‐MRS reports *metabolic flux*, enabling detection of rapid pyruvate handling changes induced by PKD1 deficiency. Although enhanced glycolysis has been described in PKD1‐deficient models (Rowe et al., 2013), flux‐based, endpoint‐quantified assessment of pyruvate metabolism using real‐time hyperpolarized MRS has not previously been examined in this targeted cellular system.

CRISPR–Cas9 gene editing provides a precise and widely validated method for targeted disruption of PKD1 (Cong et al., 2013; Doudna & Charpentier, 2014; Mali et al., 2013; Ran et al., 2013), enabling investigation of metabolic consequences in an isogenic background. Hyperpolarized [1‐^13^C]pyruvate MRS complements steady‐state assays by providing dynamic quantification of glycolytic flux (Brindle, 2015; Nelson et al., 2013), making it well suited for probing pyruvate metabolism under PC1‐deficient conditions.

A limitation of this model is that NRK‐52E kidney epithelial cells grown in 2D do not reproduce cyst architecture, epithelial curvature, lumen formation, or PC1‐dependent mechanotransduction. Therefore, the findings should be interpreted strictly as metabolic effects of PKD1 loss rather than as a complete model of cystogenesis.

The aim of this study was to determine whether PKD1 deletion alters pyruvate metabolism and glycolytic flux in NRK‐52E rat kidney epithelial cells using hyperpolarized [1‐^13^C]pyruvate MRS together with biochemical assays. These metabolic endpoints provide a foundation for future evaluation in 3D cyst cultures and in vivo ADPKD models.

## 2 MATERIALS AND METHODS

### 2.1 Cell culture

NRK‐52E cells, a male rat kidney epithelial cell line, were obtained from Merck (Sigma‐Aldrich, Copenhagen, DK). The *PKD1* gene was knocked out using CRISPR‐Cas9 technology to create the NRK‐52E *PKD1* K.O. cell line. This enables precise investigation of PC1‐deficient cellular metabolism. The NRK‐52E rat kidney epithelial cells were cultured in Dulbecco's modified Eagle's medium (DMEM) with low glucose (Sigma‐Aldrich, Copenhagen, DK) supplemented with 10% fetal bovine serum (Sigma‐Aldrich, Copenhagen, DK), 10 units/mL penicillin, and 10 mg/mL streptomycin. NRK‐52E rat kidney epithelial cells were maintained at 37°C in a humidified atmosphere (5% CO_2_) in an incubator.

Cell viability and number were determined using a trypan blue exclusion test, ensuring experimental consistency. Growth medium was changed every second or third day, and NRK‐52E rat kidney epithelial cells were sub‐cultured until reaching 80% confluence before further measurements or passaging. For each hyperpolarization experiment, approximately 20 million NRK‐52E rat kidney epithelial cells were harvested by trypsinization and resuspended in DMEM buffer.

Following harvesting, NRK‐52E rat kidney epithelial cells were used for downstream analyses. First the NRK‐52E rat kidney epithelial cells were used for hyperpolarization experiments and then the NRK‐52E rat kidney epithelial cells and medium were used for relevant RNA and enzyme analysis.

### 2.2 CRISPR‐Cas knockout of *PKD1*

Three CRISPR‐Cas sgRNAs were designed to target the coding region in exon 1 of the *PKD1* gene. Exon 1 was selected to maximize the likelihood of generating early frameshift mutations and complete functional knockout. Protospacer sequences: 5′‐ACCCGAGCAATTGACGCGGC‐3′, 5′‐TCAGCCGGGATGCGCAGACT‐3′, and 5′‐TAGGGCCAGCGCCAGGCGAG‐3′. sgRNAs were synthesized with terminal nucleotides modified with 2′‐O‐methyl 3'phosphorothioate (Hendel et al., 2015) and were acquired from Synthego (Redwood City, CA, USA). All three sgRNAs were used with SpCas9 protein (Alt‐R S.p. Cas9 Nuclease V3; IDT, Coralville, IA, USA, #1081059) and co‐delivered to cells by electroporation as described previously (Laustsen & Bak, 2019). Shortly, 6 μg Cas9 recombinant protein was complexed to 3.2 μg of each of the synthetic sgRNA by mixing and incubating at room temperature for 10 min. Following incubation, the three complexes were mixed and added to 200,000 cells in 20 μL OptiMEM (Thermo Fisher Scientific, Waltham, MA, USA, #31985062) used as electroporation buffer. The Lonza 4D electroporation device was used for nucleoporation with the settings P3, EN‐158. 5 days after nucleoporation, genomic DNA was extracted from the cells using QuickExtract (LGC Genomics GmbH, Berlin, Germany, #QE09050) following manufacturer's instructions, and the targeted *PKD1* region was PCR‐amplified using Phusion polymerase (Thermo Fisher Scientific, Waltham, MA, USA, #F566L) and primers 5′‐cacccacggccaacttggaa‐3′ and 5′‐tagtcgggccgacatccct‐3′. PCR amplicons were run on an agarose, and the bands were gel‐purified using GeneJET Gel Extraction kit (Thermo Fisher Scientific, Waltham, MA, USA, #K0692) and Sanger sequenced (Eurofins Genomics, Konstanz, Germany). The Sanger sequencing chromatogram files were analyzed for INDELs using a mock‐electroporated sample as control using the online software ICE. Following INDEL validation, cells were expanded and cryopreserved.

### 2.3 Hyperpolarization

Hyperpolarized [1‐^13^C] pyruvate (Sigma‐Aldrich, Copenhagen, DK, #490709) was polarized in a SpinAligner (Polarize, Frederiksberg, Denmark) to achieve 40%–60% solid‐state polarization. This approach, which allows a signal‐to‐noise ratio increase of >10,000 times, is critical for subsequent spectroscopic analysis (Ardenkjaer‐Larsen et al., 2003). The dissolved pyruvate solution (100 mM) was prepared in 3.4 mL of phosphate buffer (pH adjusted with NaOH). Polarization levels were measured using a Spinsolve 43 MHz benchtop NMR spectrometer (Magritek, Wellington, New Zealand).

### 2.4 Hyperpolarized carbon‐13 time‐series NMR experiments

Hyperpolarized carbon‐13 NMR experiments were performed using a Magritek Spinsolve 60 MHz Ultra system equipped with proton and carbon channels. A total of 180 acquisitions were recorded using a 10° flip angle and 2s repetition time, ensuring high temporal resolution of pyruvate and lactate dynamics.

Prior to each experiment, 20 million cells were trypsinized, centrifuged at 500 RCF for 5 min, and resuspended in 250 μL DMEM medium (without FBS and penicillin). The sample was pre‐incubated at 37°C for 5 min and then mixed with 50 μL of prediluted pyruvate dissolution solution, resulting in a final pyruvate concentration of 5 mM. This mixture was transferred to a 5‐mm NMR tube, and acquisition commenced approximately 20 s after dissolution.

Hyperpolarized [1‐^13^C]pyruvate experiments were conducted using *n* = 4 independent biological experiments for the group, each representing a separate cell culture preparation.

### 2.5 Collection of cells after hyperpolarized experiments

At the end of each time‐series experiment, the medium containing cells was transferred to an Eppendorf tube. A 10 μL aliquot was taken for cell counting to determine live and dead cells. The remaining medium was divided into two Eppendorf tubes, centrifuged at 500 RCF for 5 min, and the supernatant was removed for activity assays.

In one tube, the cell pellet was washed with 1% PBS (Sigma‐Aldrich, Copenhagen, DK), centrifuged, and the supernatant was discarded. The pellet was resuspended in lysis buffer prepared with one cOmplete™ Mini EDTA‐free Protease Inhibitor Cocktail tablet (Sigma‐Aldrich, Copenhagen, DK, #11697498001) dissolved in 10 mL Mammalian Protein Extraction Reagent (Thermo Fisher Scientific, Odense, DK, #78505), shaken for 15 min at room temperature, and centrifuged at 4°C and 14,000 rpm for 10 min. The supernatant was aliquoted into three tubes and stored at −80°C.

In the second tube, the cell pellet was resuspended in 300 μL TRI Reagent (ThermoFisher Scientific, Odense, DK, #T9424) and stored at −80°C for RNA extraction and qPCR analysis.

### 2.6 NMR data processing and kinetic analysis

Each carbon time‐series dataset was loaded into MestraNova software. Following Fourier transformation, pyruvate and lactate peaks were integrated using manually defined peak areas. Lactate/pyruvate ratios were calculated by dividing the summed integrals. All ratios were derived from fully integrated spectral peaks, ensuring quantitative consistency across time points.

In the time‐series experiments, “integral” refers to the Fourier‐transformed peak area of the lactate and pyruvate resonances, quantified using fixed regions of interest. Integrals represent signal amplitude; statistical comparisons were performed only on endpoint ratios.

### 2.7 Activity assays

Biochemical analysis of pyruvate dehydrogenase (PDH) (Sigma‐Aldrich, Copenhagen, DK, #MAK567), alanine transaminase (ALT) (Sigma‐Aldrich, Copenhagen, Dk, #MAK052), and lactate dehydrogenase (LDH) (Sigma‐Aldrich, Copenhagen, Dk, #MAK066) enzyme activities, as well as alanine (Sigma‐Aldrich, Copenhagen, Dk, #MAK001) and lactate (Sigma‐Aldrich, Copenhagen, Dk, #MAK064) concentrations, were performed using *n* = 3 independent biological experiments. The assays were performed according to the manufacturer's instructions with few alterations. In brief, cells and medium were separated by centrifugation and samples stored at −80°C until analysis.

Enzyme activity measurements were normalized to protein content, which was quantified using a Qubit 3.0 fluorometer (Fisher Scientific, Wilmington, DE). Pilot assays were conducted to establish dilution factors ensuring accurate measurements within the assay's standard curve. All analyses were performed in 384‐well plates (Sigma‐Aldrich, Copenhagen, Denmark, #CLS3702BC) using a microplate reader (SYNERGY H1, Biotek, Aarhus, Denmark).

### 2.8 RNA purification and RT‐qPCR

RNA was extracted using TRI reagent (ThermoFisher Scientific, Odense, DK) and converted to cDNA using the Maxima SYBR Green/ROX qPCR Master Mix (ThermoFisher Scientific, Odense, DK, #A46109). A melting curve analysis confirmed primer specificity, while a standard curve was generated by plotting Ct values against serial dilutions of purified PCR product. qPCR was performed on an Agilent AriaMx real‐time system. The measured levels for PKD1, PKD2, and MCT transporters were normalized to those of β‐actin, and relative gene expression was calculated using the ΔCt method. qPCR analyses were performed using *n* = 3 independent biological experiments. Each biological sample was measured in technical duplicates, which were averaged prior to statistical analysis. Utilized primers for RT‐qPCR are provided in Table 1.

**TABLE 1:** Primers for RT‐qPCR.

Transcript name	Forward primer sequences	Reverse primer sequences
β‐Actin	5′‐CCCTGTGCTGCTCAC‐3′	5′‐ACAGTGTGGGTGACCC‐3′
PKD1	5′‐TTCAGGTACCACGGCTCACG‐3′	5′‐AGGCAGGGCTGAGTCCTTTG‐3′
PKD2	5′‐GGTGCAGAGCTGGTATGTCC‐3′	5′‐TCTGGTTGGAGACTGGGTCA‐3′
MCT1	5′‐CTCTGGGCGCCGCGAGATAC‐3′	5′‐CAACTACCACCGCCCAGCCC‐3′
MCT2	5′‐GTGGCAATCATGTTCACTGG‐3′	5′‐AGAACTGGGCAACACTCCAC‐3′
MCT3	5′‐ACGGCAGGTTTCATAACAGG‐3′	5′‐ACGATAGCCATGAGCACCTC‐3′
MCT4	5′‐CCAGGCCCACGGCAGGTTCT‐3′	5′‐GCCACCGTAGTCACTGGCCG‐3′

### 2.9 Statistics

Statistical analyses of all results were performed in Prism. Independent *t*‐tests were performed between groups. Data are presented as mean ± SD. Exact *p*‐values are shown in the figures when *p* > 0.001.

## 3 RESULTS

### 3.1 *PKD1* knockout validation and cellular impact

**FIGURE 1:**
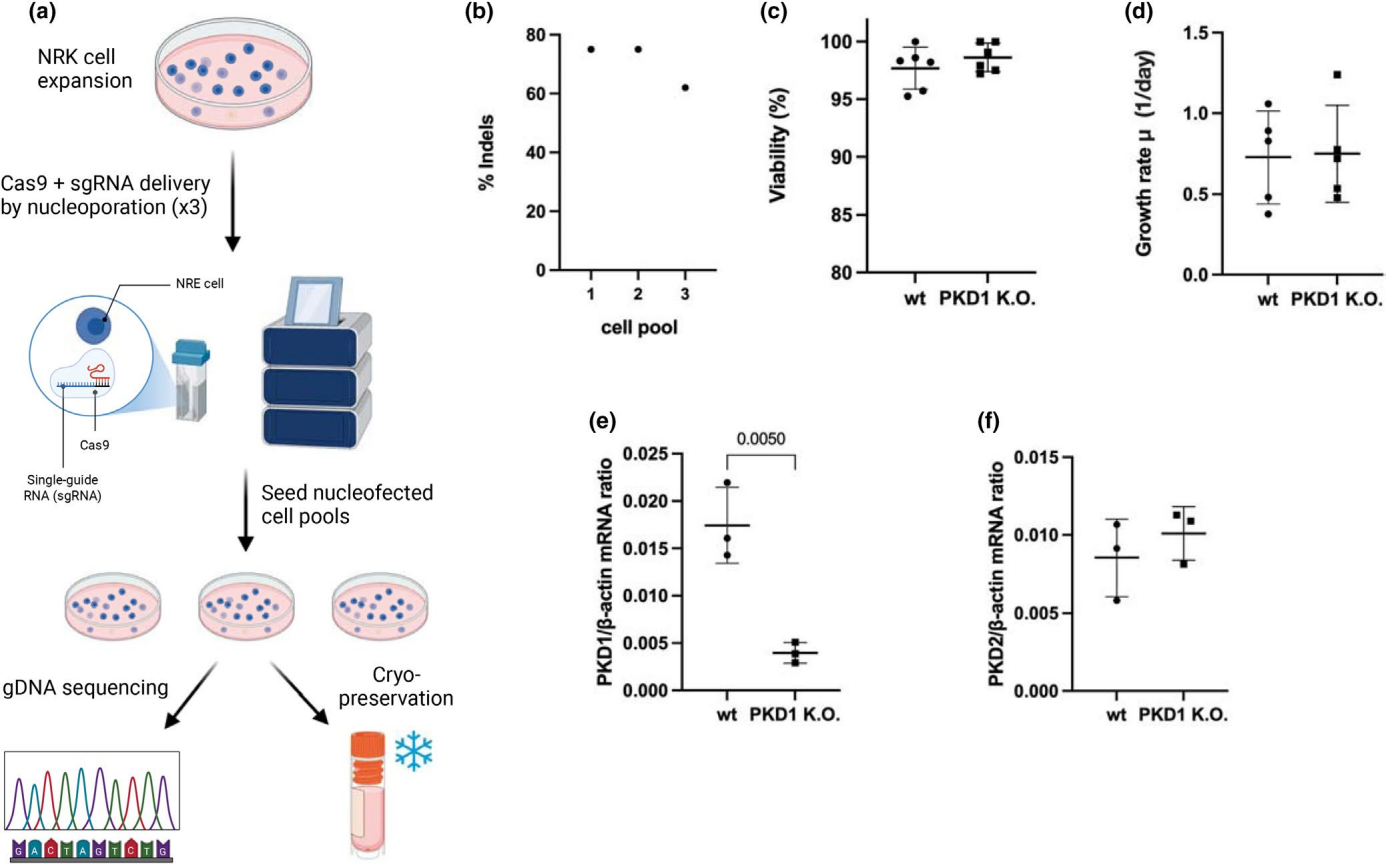
(a) Schematic overview of the CRISPR‐Cas9‐mediated K.O. strategy targeting the *PKD1* gene in NRK‐52E rat kidney epithelial cell line. Three single guide RNAs (sgRNAs) targeting exon 1 of PKD1 were complexed with Cas9 protein and delivered via nucleofection. Cells were allowed to expand, and three independent cell pools were generated and cryopreserved. Genomic DNA (gDNA) was extracted for PCR amplification and Sanger sequencing of the targeted locus. (b) Quantification of genome editing efficiency (formation of insertions/deletions; INDELs) using the ICE analysis tool. (c) Cell viabilities were assessed using Trypan Blue exclusion in wildtype (WT) and *PKD1* K.O. NRK‐52E kidney cells, Data are presented as mean ± SD from 6 independent biological experiments. (d) Growth rate (μ, per day) calculated over 48 h for WT and *PKD1* K.O. NRK‐52E kidney cells. Data are presented as mean ± SD from 5 independent biological experiments. (e) PKD1 mRNA expression measured by RT‐qPCR and normalized to β‐Actin. (f) PKD2 mRNA expression levels in WT and *PKD1* K.O. NRK‐52E kidney cells. Data in figure e and f were presented as mean ± SD from 3 independent biological experiments with technical duplicates. Statistical significance was determined by unpaired t‐test. Figure 1 was made with a licensed version of BioRender.com.

We set out to generate NRK‐52E rat kidney cells with *PKD1* knockout (K.O.). We opted for the NRK‐52E cell line, a rat kidney epithelial cell line, due to its well‐characterized epithelial origin, ease of genetic manipulation, and its relevance as a proximal tubule model—one of the primary sites affected in polycystic kidney disease. Furthermore, NRK‐52E cells exhibit stable growth and are not prone to differentiation, making them a suitable in vitro system for studying the cellular and metabolic consequences of PD1 deficiency. Figure 1a illustrates the workflow for establishing *PKD1* K.O. NRK‐52E rat kidney cells using CRISPR‐Cas technology. To ensure efficient *PKD1* K.O., three single guide RNAs (sgRNAs) targeting the coding region of rat *PKD1* exon 1 were complexed to Cas9 protein and delivered simultaneously to NRK‐52E cells in triplicates by nucleoporation. NRK‐52E cells were allowed to expand after which they were cryopreserved; genomic DNA was extracted from a subfraction of the three cell populations, and the targeted *PKD1* locus was PCR‐amplified and sequenced. Sequencing results were analyzed by the online software ICE to evaluate editing outcomes and the frequency and distribution of DNA insertions/deletions (INDELs). These data showed potent INDEL formation with frequencies ranging from 62% to 75% (Figure 1b). To further confirm *PKD1* knockout we purified mRNA from the three cell populations and analyzed *PKD1* mRNA expression by RT‐qPCR, which confirmed significantly reduced *PKD1* transcript levels in *PKD1* K.O. NRK‐52E kidney cells compared to wildtype (WT) NRK‐52E kidney cells, confirming the effectiveness of the knockout (Figure 1e). Gene expression values are presented as β‐actin–normalized ΔCt values to preserve direct comparison of absolute transcript levels between groups. PKD2 mRNA expression did not differ significantly from WT (Figure 1f). Cell viabilities (Figure 1c), and growth rates (Figure 1d) remained unaffected, indicating that PKD1 knockout did not alter viability or proliferation under the tested conditions.

### 3.2 Lactate production and glycolytic flux in *PKD1* K.O. NRK‐52E kidney cells

**FIGURE 2:**
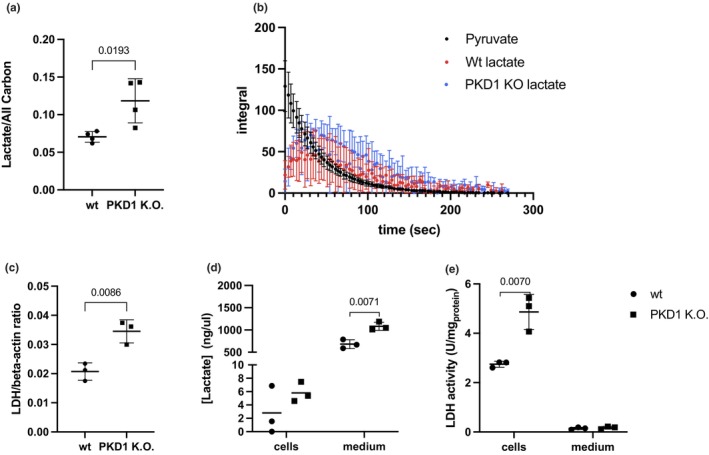
(a) The intracellular lactate‐to‐total carbon ratio was quantified in wildtype (WT) and *PKD1* K.O. NRK‐52E kidney cells using hyperpolarized MRS, with data presented as mean ± SD from 4 independent biological experiments (b) Time‐resolved kinetic curves depicting real‐time were generated for the two different cell types. Integral denotes FT peak area (arbitrary units) for lactate and pyruvate. (c) LDH (lactate dehydrogenase) gene expression levels were assessed by RT‐qPCR and normalized to β‐Actin, data was presented as mean ± SD from 3 independent biological experiments with technical duplicates. (d) Total lactate concentrations were measured in both the cellular and extracellular (medium) fractions after the hyperpolarized experiment. (e) LDH enzymatic activity was measured in cell lysates and culture medium, Data in figure d and e was presented as mean ± SD from 3 independent biological experiments. Statistical significance was determined by unpaired *t*‐test.

To investigate the metabolic consequences of *PKD1* K.O., we focused on lactate metabolism. We first measured the lactate‐to‐total carbon ratio in *PKD1* K.O. and WT NRK‐52E kidney cells using hyperpolarized magnetic resonance spectroscopy (HP‐MRS) with [1‐^13^C] pyruvate. The real‐time metabolite levels were calculated from *PKD1* K.O. and WT NRK‐52E kidney cells and normalized to total cellular carbon content. *PKD1* K.O. kidney cells exhibited a significantly higher lactate‐to‐carbon ratio compared to WT NRK‐52E kidney cells (Figure 2a,b). Figure 2b shows time‐resolved lactate signal integrals (HP‐MRS) during the experiment. Group trajectories are displayed descriptively; primary inference relies on endpoint/summary measures.

The Lactate/All Carbon ratio shown in Figure 2a represents an endpoint summary derived from the fully integrated acquisition window shown in Figure 2b, rather than a single time point, while the time‐resolved curves are presented to illustrate dynamic behavior.

To assess whether altered lactate levels were accompanied by changes in lactate dehydrogenase (LDH), we analyzed LDH expression. LDH is the enzyme catalyzing the reversible conversion of pyruvate to lactate—the terminal step of glycolysis. *LDH* gene expression was measured by RT‐qPCR and normalized to β‐actin.

To further assess lactate production, we measured steady‐state lactate concentrations in both the cell lysates and media. While levels were overall low in the cell lysates, PKD1 K.O. NRK‐52E kidney showed a significant upregulation of LDH transcripts (Figure 2c) and significantly higher LDH enzymatic activity in cell lysates (Figure 2e). Lactate in culture medium was significantly higher (Figure 2d).

The coordinated increase in LDH transcription, enzymatic activity, and lactate production supports an enzymatically driven enhancement of glycolytic flux in PKD1‐deficient NRK‐52E kidney cells.

### 3.3 Altered monocarboxylate transporter (MCT) expression

**FIGURE 3:**
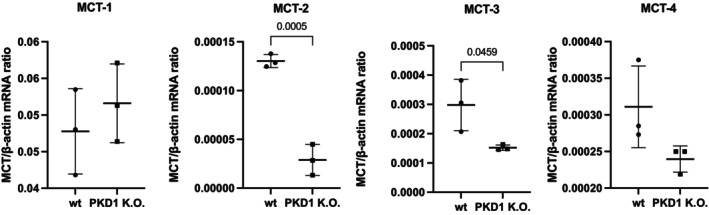
Relative mRNA expression levels of MCT1, MCT2, MCT3, and MCT4 were analyzed in wildtype (WT) and *PKD1* K.O. NRK‐52E cells by RT‐qPCR. Expression levels were normalized to the housekeeping gene β‐Actin. Data were presented as mean ± SD from 3 independent biological experiments with technical and statistical significance was determined by unpaired t‐test.

To further characterize the metabolic phenotype associated with PC1 deficiency, we assessed the mRNA expression of MCTs, a family of membrane proteins responsible for the bidirectional transport of key metabolic intermediates such as lactate, pyruvate, and ketone bodies. These transporters play essential roles in maintaining intracellular pH and supporting glycolytic flux, particularly under conditions of increased metabolic demand. MCT1 expression did not differ significantly, whereas MCT2 and MCT3 were significantly downregulated. MCT4 displayed a downward trend that did not reach statistical significance, suggesting potential isoform‐specific regulation (Figure 3).

### 3.4 Alteration of alanine and pyruvate metabolism in *PKD1* K.O. NRK‐52E kidney cells

**FIGURE 4:**
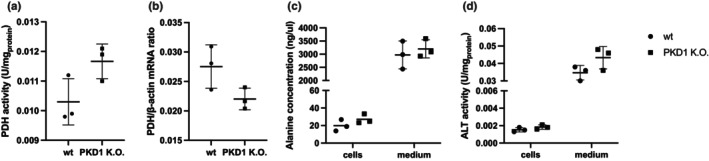
(a) Pyruvate dehydrogenase complex (PDH) enzymatic activity was assessed in wildtype (WT) NRK‐52E cells and *PKD1* K.O. (b) Total PDH complex mRNA expression levels were assessed and normalized to β‐Actin. (c) Intracellular alanine concentrations were measured. (d) Alanine transaminase (ALT) enzymatic activity was assessed in cell lysates using a colorimetric assay. Data are presented as mean ± SD and *n* = 3.

We examined the activity of the pyruvate dehydrogenase (PDH) complex, which catalyzes the conversion of pyruvate into acetyl‐CoA. PDH enzymatic activity and PDH mRNA expression did not differ significantly between groups (Figure 4a,b). Alanine concentrations and ALT activity did also not differ significantly between PKD1 K.O. and WT NRK‐52E kidney cells (Figure 4c,d).

## 4 DISCUSSION

The present study demonstrates that PKD1 deficiency in NRK‐52E rat kidney epithelial cells is associated with statistically significant alterations in glycolytic flux, evidenced by higher pyruvate‐to‐lactate conversion using hyperpolarized [1‐^13^C]pyruvate MRS and increased extracellular lactate, alongside significantly elevated LDH expression and enzymatic activity. These findings align with prior observations that PC1 deficiency promotes a shift toward glycolysis (Rowe et al., 2013) and support the use of HP‐MRS as a sensitive, flux‐level readout of real‐time metabolic remodeling (Brindle, 2015; Rodrigues et al., 2014). Importantly, this study extends previous cellular PKD research by demonstrating *flux‐level* metabolic alterations in a controlled NRK‐52E cell model rather than changes in steady‐state metabolite pools alone.

This glycolytic phenotype is conceptually consistent with the Warburg effect—that is, preferential glycolysis despite oxygen availability (Vander Heiden et al., 2009) and with renal pseudohypoxia described in diabetic kidney disease (Brownlee, 2001; Laustsen et al., 2013, 2014, 2019; Palm et al., 2004). In our model, the dominant change is an increased pyruvate‐to‐lactate conversion; by contrast, PDH activity and PDH transcripts did not differ significantly, indicating that mitochondrial pyruvate oxidation remained unchanged under the present experimental conditions. Accordingly, while the data are consistent with a glycolytic shift, they do not provide evidence for altered mitochondrial pyruvate oxidation under the present conditions.

Beyond lactate, we assessed the alanine transamination branch. Alanine levels and ALT activity did not differ significantly between groups. Together, these endpoints suggest that lactate production—not alanine formation—is the predominant fate of pyruvate under PKD1 deficiency in this system, which is consistent with broader reports of metabolic remodeling in PKD models (Padovano et al., 2018). Future studies with increased sample size and direct flux tracing into the TCA cycle will be needed to resolve subtle changes in alternative pyruvate fates.

Monocarboxylate transporter (MCT) expression provided further context. We observed no significant change in MCT1, whereas MCT2 and MCT3 were significantly downregulated, and MCT4 trended lower. The increased lactate accumulation despite reduced or unchanged MCT transcripts can be explained by factors beyond transcription: MCT function and lactate export can be regulated post‐transcriptionally (protein abundance, membrane localization), by pH gradients, and by metabolite‐driven kinetics (Halestrap, 2012). Moreover, lactate handling occurs within a broader metabolic shuttle framework (Chapman et al., 2015; Draoui & Feron, 2011). Transcriptional programs such as HIF‐1α, MYC, and AMPK (Kobayashi et al., 2021; Song et al., 2024) may also influence MCT regulation in ways not reflected at the mRNA level. These considerations motivate protein‐level quantification (immunoblotting, transport assays) and functional readouts of MCT activity in future work. Future studies assessing MCT protein abundance and transporter activity could be important to resolve the divergence between transcript levels and lactate accumulation.

From a modeling perspective, our NRK‐52E monolayer system prioritizes mechanistic clarity for PKD1‐driven metabolic flux, while recognizing that ADPKD pathogenesis in vivo typically involves segment specificity (distal nephron/collecting duct) and a second‐hit process over decades. Gene‐editing approaches to model PKD1 deficiency are widely used across cell systems, including iPSC‐based models (Romano et al., 2020), underscoring the translational relevance of targeted knockouts for dissecting mechanism. At the tissue level, additional microenvironmental stressors (e.g., crystal deposition) can accelerate cystogenesis (Torres et al., 2019); integration of such factors with metabolic imaging may help connect cellular metabolic states to cyst initiation and growth.

Clinically and translationally, our results support hyperpolarized [1‐^13^C]pyruvate MRS as a biomarker platform for detecting glycolytic remodeling relevant to PKD. While the present data are in a cell model, HP‐[1‐^13^C]pyruvate has been used to monitor renal metabolic changes and pharmacologic perturbations in vivo (Laustsen et al., 2013, 2019; Qi et al., 2018). Future studies may evaluate flux‐sensitive HP‐MRS endpoints (e.g., lactate labelling, k_PL) evaluated in 3D cyst cultures and animal models that capture segment specificity and second‐hit biology, enabling assessment of disease progression and treatment response with a mechanistic anchor.

Given that HP‐pyruvate MRS is already validated for detecting renal metabolic changes in vivo, these findings highlight a clear translational path toward evaluating PKD‐associated metabolic remodeling in animal models.

## 5 LIMITATIONS

This study has limitations, including the use of a 2D NRK‐52E cell monolayer model that does not reproduce 3D cyst architecture or nephron‐segment specificity. Second, sample size (*n* = 3 per group) limits power for secondary endpoints (PDH, alanine/ALT), and time‐series trajectories are presented descriptively without repeated‐measures inference. Third, protein‐level confirmation was not performed for all targets (e.g., MCT isoforms). These constraints limit interpretation of secondary endpoints.

Nevertheless, the consistency across metabolic, enzymatic, and transcriptional endpoints strengthens the overall interpretation.

In summary, PKD1 deficiency in NRK‐52E rat kidney epithelial cells is associated with significant enhancement of glycolytic flux and extracellular lactate accumulation, supported by increased LDH expression and activity. Hyperpolarized [1‐^13^C]pyruvate MRS provides a sensitive, real‐time window into these metabolic shifts and represents a promising biomarker strategy to be tested in cyst‐forming systems and in vivo (Laustsen et al., 2013, 2019; Qi et al., 2018).

## AUTHOR CONTRIBUTIONS

L.B.B. and C.L. conceived and designed research. I.K.M., E.A., S.R.D., and L.B.B. performed experiments. I.K.M., R.O.B., and L.B.B. analyzed data. I.K.M., C.L., R.O.B. and L.B.B. interpreted results of experiments. R.O.B. and L.B.B. prepared figures. I.K.M. and L.B.B. drafted manuscript. C.L., E.A., S.R.D., R.O.B., and L.B.B. edited and revised manuscript. I.K.M., C.L., E.A., S.R.D., R.O.B., and L.B.B. approved final version of manuscript.

## FUNDING INFORMATION

L.B.B. and C.L. gratefully acknowledge support from a Lundbeck Foundation (R272‐2027‐4023), Novo Nordisk Foundation (0077911), The Danish Cancer Society (R279‐A16251), and Karen Elise Jensens Foundation (500043). R.O.B. gratefully acknowledges support from a Lundbeck Foundation Fellowship (R238‐2016‐534 3349) and Innovation Fund Denmark (8056‐00010B).

## CONFLICT OF INTEREST STATEMENT

C.L. is an inventor on patents and patent applications related to d‐DNP and metabolic imaging. R.O.B. holds equity in Kamau Therapeutics and UNIKUM Therapeutics and is a cofounder of UNIKUM Therapeutics. Neither company was involved in the present study. R.O.B. is an inventor on patents and patent applications related to CRISPR/Cas and cellular therapies. R.O.B. reports research funding from Novo Nordisk.

## ETHICS STATEMENT

This study does not report experiments carried out on animals, human subjects, or human fetal tissue. All experiments were performed using the established NRK‐52E rat kidney epithelial cell line.

## Data Availability

Data will be made available upon reasonable request.
